# Endoplasmic Reticulum Stress Signaling in Plant Immunity—At the Crossroad of Life and Death

**DOI:** 10.3390/ijms161125964

**Published:** 2015-11-05

**Authors:** Camilla J. Kørner, Xinran Du, Marie E. Vollmer, Karolina M. Pajerowska-Mukhtar

**Affiliations:** Department of Biology, University of Alabama at Birmingham, 1300 University Blvd., Birmingham, AL 35294, USA; ckoerner@uab.edu (C.J.K.); xinrandu@uab.edu (X.D.); mvollmer@uab.edu (M.E.V.)

**Keywords:** plant immune responses, IRE1, programmed cell death, ER stress signaling, Arabidopsis

## Abstract

Rapid and complex immune responses are induced in plants upon pathogen recognition. One form of plant defense response is a programmed burst in transcription and translation of pathogenesis-related proteins, of which many rely on ER processing. Interestingly, several ER stress marker genes are up-regulated during early stages of immune responses, suggesting that enhanced ER capacity is needed for immunity. Eukaryotic cells respond to ER stress through conserved signaling networks initiated by specific ER stress sensors tethered to the ER membrane. Depending on the nature of ER stress the cell prioritizes either survival or initiates programmed cell death (PCD). At present two plant ER stress sensors, bZIP28 and IRE1, have been described. Both sensor proteins are involved in ER stress-induced signaling, but only IRE1 has been additionally linked to immunity. A second branch of immune responses relies on PCD. In mammals, ER stress sensors are involved in activation of PCD, but it is unclear if plant ER stress sensors play a role in PCD. Nevertheless, some ER resident proteins have been linked to pathogen-induced cell death in plants. In this review, we will discuss the current understanding of plant ER stress signaling and its cross-talk with immune signaling.

## 1. Introduction

In order to defend themselves against diseases, plants rely on early recognition of pathogens followed by fast responses to eliminate the attacker. The plant immune system contains several layers [1]. The basal layer of defense is activated by the presence of Microbe-Associated Molecular Patterns (MAMPs) in the apoplast. This layer of defense is referred to as MAMP Triggered Immunity (MTI) and protects the plant against non-specialized microorganisms. MAMPs are perceived by plasma membrane receptors, of which some dependent on maturation and ER protein folding quality control [2,3,4,5]. Another ER-dependent basal defense response is the secretion of anti-microbial proteins to the apoplast [6]. Both MAMP receptors and secreted anti-microbial proteins pass through the ER, and high protein folding and secretion capacity of the ER is crucial for rapid and effective basal immune responses [7]. However, specialized pathogens can suppress basal defense responses through pathogen-encoded effector proteins. This leads to Effector Triggered Susceptibility (ETS), allowing the pathogen to proliferate inside the plant and cause disease [1]. However, in some cases specific effectors are directly or indirectly recognized by plant R proteins. This recognition activates a second and more robust layer of immune response, termed Effector Triggered Immunity (ETI) [1]. In many cases, ETI leads to programmed cell death (PCD), often also referred to as Hypersensitive Response (HR), trapping the pathogens inside a region of dead cells. Over the last decade, it has become clear the ER and the regulation of its capacity play an important role in immune signaling. In this review, we will present the current understanding of ER stress signaling in plants and highlight the cross-talk between the ER stress signaling pathways and immune responses.

## 2. Laying out the Road Map—ER Stress Signaling Pathways in Plants

### 2.1. Inositol Requiring Enzyme 1 (IRE1): The Conserved ER Stress Sensor

**Figure 1:**
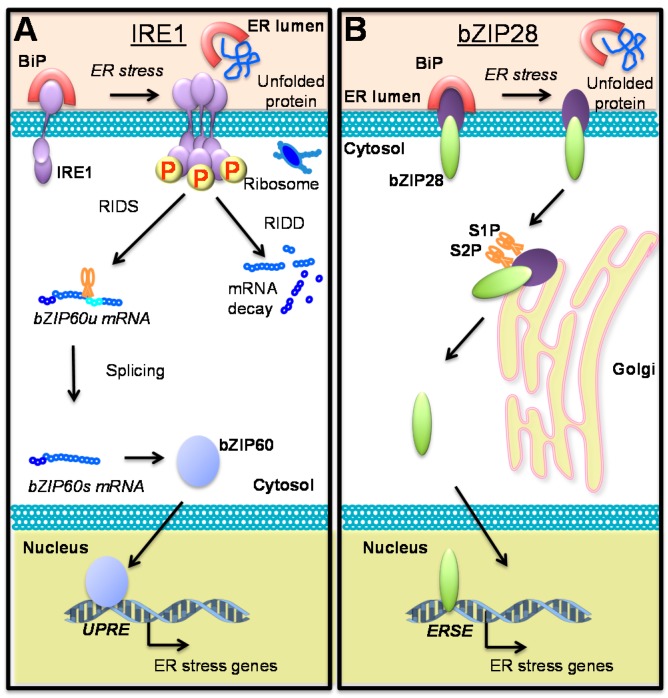
Pathways regulating ER stress responses in Arabidopsis. (**A**,**B**) The ER-localized domains of the two conserved ER stress sensors Inositol Requiring Enzyme 1 (IRE1) and Basic Leucine Zipper 28 (bZIP28) interact with the heat shock protein Binding Protein (BiP). Accumulation of unfolded proteins leads to BiP disassociation from the ER stress sensors (**A**,**B**). Plant IRE1 is predicted to undergo oligomerization followed by autophosphorylation of the cytosolic kinase domains. Activation of the IRE1 ribonuclease domain leads to splicing of Basic Leucine Zipper 60 (bZIP60) mRNA and bulk degradation of selected mRNAs through Regulated IRE1-Dependent Decay (RIDD). Spliced bZIP60 mRNA is translated to an active transcription factor, which moves to the nucleus and up-regulates genes containing Unfolded Protein Response Element (UPRE) *cis*-elements in their promoters (**A**); Unchaperoned bZIP28 moves to the Golgi, where it is proteolytically cleaved by Site 1- and Site 2-transcription factor Peptidases (S1P and S2P), releasing the N-terminal fragment of bZIP28 containing the bZIP DNA-binding domain. bZIP28 controls the expression of its target genes through binding to Endoplasmic Reticulum Stress Element (ERSE) *cis-*elements in their promoter (**B**). P—protein phosphorylation, AA—amino acid.

IRE1 was first identified as a transcriptional regulator of ER genes in yeast [8] and later its homologs were identified in animals and plants [9,10], all sharing a conserved mode of action. IRE1 is a transmembrane receptor protein located in the ER membrane with a cytosolic kinase/ribonuclease domain and a luminal ER stress sensor domain [11]. The *Arabidopsis thaliana* (hereafter: Arabidopsis) genome encodes two *IRE1* homologs, *IRE1a* and *IRE1b*, playing both overlapping and specific roles. While the activation mechanism of plant IRE1 has not been shown, it was demonstrated in yeast and animals that the ER lumen-localized heat shock protein Binding Protein (BiP) binds to the luminal domain of IRE1 under unstressed conditions. The binding of BiP desensitizes of the IRE1 sensor domain, hereby blocking IRE1 self-activation. Upon ER stress, BiP disassociates from IRE1 and subsequently binds to misfolded/unfolded proteins (Figure 1A) and releases IRE1 to activate its downstream components. A similar IRE1 activation mechanism has been proposed for plant IRE1 [12,13]. Although the activation of IRE1 is believed to be generally controlled by BiP, emerging evidence from yeast and animals systems has shown that IRE1 can be activated by directly binding to unfolded proteins in the ER lumen or by changes in membrane lipid composition [14,15,16,17]. Whether these mechanisms also contribute to IRE1 activation in plants is not known. Activation of IRE1 leads to its oligomerization, followed by autophosphorylation of the cytosolic kinase domain [18,19] that further activates the ribonuclease domain [18]. The active IRE1 ribonuclease domain has been shown to splice specific target mRNAs, such as X-box Binding Protein1 (XBP1) in animals, HAC1 in yeast, *At*bZIP60 in Arabidopsis and *Os*bZIP50 in rice [20,21] in a process termed Regulated IRE1-Dependent Splicing (RIDS). The unconventional splicing of the two known plant IRE1 mRNA targets gives rise to mRNAs that can be translated into functional transcription factors. Both *At*bZIP60 and *Os*bZIP50 regulate known ER stress marker genes encoding proteins directly involved in alleviating ER stress (Figure 1A) [20,22]. It has been demonstrated that *At*bZIP60 binds to Unfolded Protein Response Elements (UPRE), *cis*-elements found in the promoters of many genes induced by ER stress [23]. Activated IRE1 can also perform bulk degradation of specific mRNAs in a process termed Regulated IRE1-Dependent Decay (RIDD) [24,25,26]. It has been suggested that during RIDD IRE1 degrades mRNAs encoding proteins that require ER folding, thereby reducing the burden on the ER protein folding machinery [24].

### 2.2. The ER Membrane-Anchored Transcription Factor Basic Leucine Zipper 28 (bZIP28) also Controls ER Stress Signaling

The ER stress sensor bZIP28, homologous to Activating Transcription Factor 6 (ATF6) in animals, represents another branch of the ER stress signaling pathway in plants (Figure 1B). Like IRE1, bZIP28 is also activated through stress-induced accumulation of misfolded proteins in the ER lumen. The full length bZIP28 is a transmembrane protein anchored to the ER membrane by a type II transmembrane domain. bZIP28 contains a C-terminal tail facing the ER lumen and a N-terminal cytosolic DNA binding domain [27,28]. BiP has been shown to bind to the C-terminal tail of bZIP28 under unstressed conditions. However, BiP disassociates from bZIP28 as unfolded proteins build up under ER stress conditions [29]. Unchaperoned bZIP28 protein translocates from ER to Golgi, where it is proteolytically processed by Site 1- and Site 2-transcription factor Peptidases (S1P, S2P) (Figure 1B) [27]. The cleaved form of bZIP28 containing the DNA binding domain relocates to the nucleus and regulates the transcription of ER stress genes carrying the Endoplasmic Reticulum Stress Element (ERSE) *cis*-regulatory motifs (Figure 1B) [12,27]. Although there are some overlaps between genes transcriptionally regulated by bZIP60 and bZIP28, both pathways have unique targets [23]. Interestingly, the Arabidopsis genome encodes a second transcription factor processed by S1P and S2P, namely *Basic Leucine Zipper 17* (*bZIP17*) [30]. While bZIP17 is not involved in ER stress signaling induced by the chemical tunicamycin, it plays a crucial role in responses to salt stress and abscisic acid as well as brassinolides signaling [30,31,32]. However, it remains unknown whether bZIP17 is activated by biotic stresses. Interestingly, it was recently reported that SA-induced bZIP60 splicing is reduced in the *bzip17* mutant background suggesting some cross-talk between bZIP17 and the IRE1 branch of the ER stress signaling pathway [33].

### 2.3. Novel Plant ER Stress Signaling Regulators

**Figure 2:**
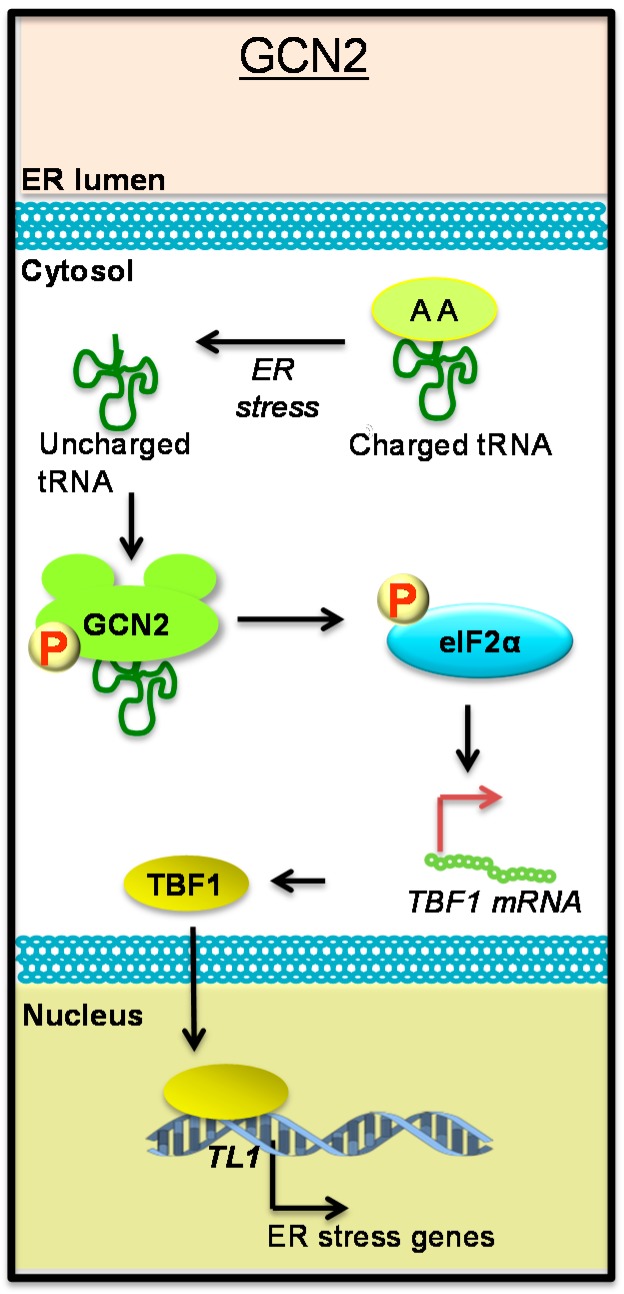
Novel ER stress regulator GCN2 links defense and ER stress gene expression. At high concentrations of uncharged tRNAs Eukaryotic Translation Initiation Factor 2 alpha (eIF2α) is phosphorylated by General Control Nonderepressible (GCN2). Phosphorylated eIF2α controls translation of several mRNAs, including *TL1*-Binding Transcription Factor 1 (TBF1) that in turn induces expression of ER resident genes following an immune challenge. GCN2 is also activated by phytohormones Salicylic Acid (SA) and Jasmonic Acid (JA). P—protein phosphorylation, AA—amino acid.

Animal cells encode a third ER membrane anchored ER stress sensor, PKR-like Eukaryotic Initiation Factor 2a Kinase (PERK). PERK senses ER stress in a manner analogous to IRE1 and bZIP28/ATF6, but their downstream outputs are distinct. While IRE1 and bZIP28/ATF6 activation leads to an induction in gene expression, active PERK phosphorylates α subunit of Eukaryotic Translation Initiation Factor 2 (eIF2α), resulting in repression of translation [34]. No PERK homologues have been identified in plant genomes to date. However, the Arabidopsis genome encodes a functionally equivalent protein named General Control Nonderepressible 2 (GCN2). GCN2 is a cytosolic serine/threonine-protein kinase that also phosphorylates eIF2α [35,36]. The GCN2 pathway is intriguing because it is not activated through the accumulation of unfolded/misfolded proteins in the ER; instead, GCN2 activation depends on amino acid availability [37]. During amino acid deprivation, uncharged tRNAs accumulate and their binding to GCN2 activates its kinase domain leading to trans-autophosphorylation, and subsequent phosphorylation of eIF2α and alterations in translation activity [37] (Figure 2). Interestingly, Arabidopsis eIF2α is phosphorylated by GCN2 upon treatment with defense hormones Salicylic Acid (SA) and Jasmonic Acid (JA) [38]. Recently, it was reported that β-aminobutyric acid (BABA), a priming agent that provides broad-spectrum disease protection in plants, acts through GCN2-dependent phosphorylation of eIF2α [39]. However, unlike in animals, virus infection does not induce phosphorylation of eIF2α in plants [36]. GCN2-dependent eIF2α phosphorylation in turn controls the translation of the transcription factor *TL1*-Binding Transcription Factor 1 (TBF1), also known as Heat Shock Factor 4 (HSF4) and Class B Heat Shock Factor 1 B (HSFB1) [40], which is a protein involved in the upregulation of ER stress marker genes upon SA treatment (see below) [41,42]. GCN2 has also been shown to be involved in growth and developmental processes [43].

Two NAC (NAM, ATAF, CUC) transcription factors were recently identified as regulators of ER stress responses, namely NAC089, also known as Fructose Sensing Quantitative trait locus 6 (FSQ6) and NAC062, also known as NTM-like 6 (NTL6) [44,45]. Both NAC089 and NAC062 are transmembrane proteins tethered in the ER and plasma membrane, respectively. Both proteins undergo proteolytic cleavage and translocate to the nucleus after induction of ER stress [44,45]. NAC062 was previously linked to cold stress and immunity, including the regulation of *PR* genes expression during cold stress [46]. While the ER stress-induced transcriptional upregulation of NAC089 depends on both the bZIP28 and the IRE1/bZIP60 pathway, NAC062 transcriptional regulation is exclusively controlled by IRE1/bZIP60 [44,45].

The β subunit Arabidopsis GTP Binding Protein (AGB1) of the plant G protein complex is another example of a signaling protein linked to both ER stress signaling [47,48] and immune responses [49,50,51,52,53,54]. G protein complexes are membrane-associated complexes, which in their active state regulate the activity of client proteins [55]. However, it is not clear if AGB1 directly perceives ER stress and if so, how. It is also not known if the downstream targets of AGB1 represent novel or known regulators of ER stress and immune signaling. Interestingly, lost-of-function mutants of several other G-protein subunits interacting with AGB1 have also been shown to be more susceptible to bacterial pathogens [50,56] and more sensitive to the ER stress inducing chemical tunicamycin [57], further supporting the notion that G protein signaling is involved in both ER stress and immune signaling.

## 3. Traffic on the Highway—ER Stress Signaling and Biotrophic Pathogens

### 3.1. Defense Hormone Salicylic Acid Activates ER Stress Signaling

The link between SA and ER stress signaling was originally established based on the observation that in Arabidopsis the application of SA leads to drastic changes in the expression of many genes encoding ER resident proteins important for protein folding and secretion [7]. This observation led to the hypothesis that SA primes the ER capacity to assist in the production and secretion of defense proteins [7]. Enhanced susceptibility to *Pseudomonas syringae* in several of these ER protein folding and secretion mutants supports this hypothesis [7]. It was later shown that some of these ER genes are transcriptionally regulated by transcription factor TBF1 and this regulation is genetically dependent on a key SA signaling regulator Nonexpressor of *PR* Genes 1 (NPR1) [40]. The fact that only selected ER genes were regulated by TBF1 suggests the existence of a second branch regulating SA-induced ER marker genes. Consistent with this observation, it was recently shown that SA treatment induces the splicing of *At*bZIP60 and *Os*bZIP50, the hallmark of IRE1 activation [33,58,59,60] linking activation of an ER stress sensor to defense responses in both Arabidopsis and rice. Arabidopsis *ire1a* mutants were furthermore shown to be more susceptible to the hemibiotrophic pathogen *Pseudomonas syringae* pv. *maculicola* [60] suggesting that IRE1a is a positive regulator of SA-mediated defense responses in Arabidopsis.

### 3.2. Induction of ER Stress by Virus Infection

In addition to bacterial pathogens, plant-infecting viruses can also activate the ER stress signaling mechanism. During infection, viruses hijack the cellular machinery to replicate their genomes and translate viral proteins. In animal systems, virus infections have long been known to induce ER stress markers and specific viral proteins have been found to directly manipulate ER stress signaling pathway components to enhance viral propagation [61]. Recently, similar observations were made in plants. In Arabidopsis, a broad range of viruses induce the gene expression of ER stress marker genes such as BiPs and Calreticulins (CRTs) [62,63,64,65]. Moreover, spliced bZIP60 mRNA accumulates in Arabidopsis infected with potyvirus *Turnip mosaic virus* (TuMV) [66]. ER stress marker gene expression is also induced in *Nicotiana benthamiana* (*N. benthamiana*) infected with potexvirus *Potato virus X* (PVX) and fijivirus *Rice black-streaked dwarf virus* (RBSDV) [67,68], but not with tobamovirus *Tobacco mosaic virus* (TMV) [69]. The upregulation of ER genes has been linked to specific viral proteins: TuMV 6K2, PVX TGBp and RBSDV P10 [66,67,68], all of which are ER-associated proteins. Both *N. benthamiana* *bzip60* knock-down plants and Arabidopsis *bzip60* knock-out mutants have lower viral titers of PVX and TuMV [66,67], suggesting that ER stress activation act as a positive regulator of virus replication. However, the ER localized Movement Protein (MP) of TMV interacts with Cell Division Cycle 48 (CDC48), a key cellular regulator of degradation of polyubiquitinated proteins during ER stress. The interaction between TMV MP and CDC48 promotes the degradation of TMV MP [70], implying that not all plant viruses would benefit from inducing ER stress signaling.

### 3.3. Plant Pathogen Effectors Target ER Localized Proteins

Plant pathogens have the ability to reprogram the cellular function of their hosts by injecting effector proteins directly into the host cell. Such effector proteins often target host proteins involved in defense [71] but several effectors from diverse plant pathogens were shown to target ER resident proteins [72,73,74]. One example is the effector protein HopD1 from *Pseudomonas syringae* pv. *tomato* DC3000 that interacts with Arabidopsis ER resident protein NTM1-like 9 (NTL9) [73]. One study found that NTL9 is a negative regulator of basal defense and *ntl9* mutants are more resistant to *Pseudomonas syringae* pv. *tomato* DC3000 [75]. However, a different study found that the susceptibility of *ntl9* mutants to *Pseudomonas syringae* pv. *maculicola* do not differ from wild type plants when plants were pressure infiltrated with a needleless syringe [76]. The same study, however, found that NTL9 controls the expression of SA biosynthesis gene *Isochorismate Synthase 1* (*ICS1*) in guard cells and NTL9 is needed for stomatal closure during pathogen infection [76]. NTL9 is an ER membrane-tethered transcription factor that is believed to undergo proteolytic processing under specific conditions. However, the protease cleaving NTL9 remains to be identified and whether NTL9 processing can be linked to ER stress is currently unknown. The Pi03192 effector from oomycete *Phytophthora infestans* is an example of another effector targeting ER membrane-tethered transcription factors. Pi03192 directly interacts with two potato NAC Targeted by *Phytophthora* (NTP) transcription factors, NTP1 and NTP2 [74]. Interestingly, the closest Arabidopsis homolog of NTP1 is NAC062, a membrane anchored transcription factor linked to ER stress signaling and immunity (see above) [44,77].

## 4. Turning down a Dead End Street—ER Stress and Programmed Cell Death in Metazoans

Previously, the mode of action of ER stress sensors in pro-survival signaling has been described in detail. The following part of this review focuses on their potential pro-death roles in animals. Additionally, ER-dependent cell death mechanisms will be summarized.

### 4.1. Core Regulators and Executors of PCD

**Figure 3:**
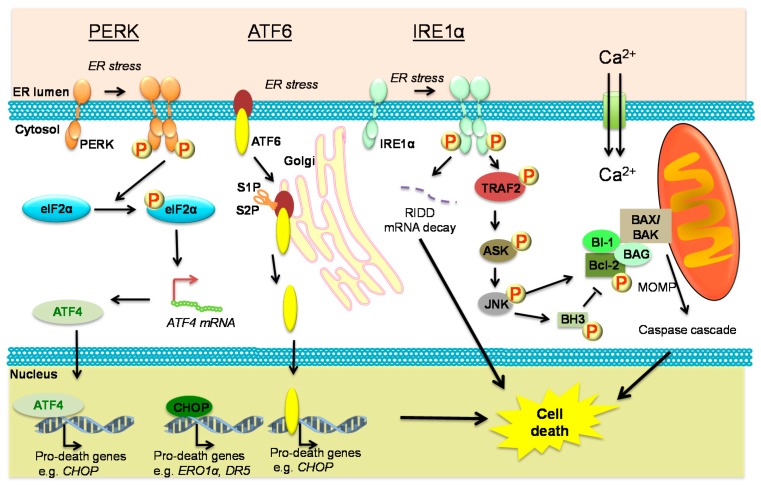
ER stress sensors and Programmed Cell Death in animals. Severe ER stress activates PKR like Eukaryotic Initiation Factor 2a Kinase (PERK) leading to phosphorylation of Eukaryotic Translation Initiation Factor 2 α (elF2α) and translational upregulation of Activating Transcription Factor 4 (ATF4), a transcription factor positively regulating several pro-death genes, including C/EBP Homologous Protein (CHOP). Activating Transcription Factor 6 (ATF6) is proteolytically processed in the Golgi and an active transcription factor is released. ATF6 also up-regulates pro-death genes. Activated Inositol Requiring Enzyme 1 α (IRE1α) can promote cell death through bulk degradation of selected mRNA through Regulated IRE1-Dependent Decay (RIDD) or by phosphorylating Tumor Necrosis Factor Receptor Associated Factor 2 (TRAF2) and activating the downstream pathway, including activation of BCL-2 Homology Domains (BH3) and Bcl-2-Associated X/Bcl-2-Antagonist Killer (BAX/BAK)-regulated Mitochondrial Outer Membrane Permeabilization (MOMP) and caspase cascade-dependent cell death. P—protein phosphorylation.

Three distinct types of PCD have been described in animals: autophagy, necrosis and apoptosis, with the latter being by far the best understood pathway. While many different stimuli can trigger PCD through distinct upstream signaling, undeniably the activation of caspases is at the core of committed cell death signaling in animals. [78]. One highly conversed caspase activation mechanism relies on the release of cytochrome C from mitochondria in a process termed Mitochondrial Outer Membrane Permeabilization (MOMP). MOMP is positively regulated by Bcl-2-Associated X/Bcl-2-Antagonist Killer (BAX/BAK), which are kept inactive by physically interacting with specific members of the B-cell lymphoma 2 (Bcl-2) family. A conserved positive regulator of cell death also belonging to the Bcl-2 family found throughout the animal kingdom is BCL-2 Homology Domains (BH3), which acts as a negative regulator of Bcl-2 (Figure 3) [78].

### 4.2. ER Stress Signaling and PCD

Interestingly, ER stress sensors known to be involved in pro-survival ER stress signaling have also been implicated in cell death signaling. Upon stress, PERK is activated and phosphorylates eIF2α, which aims to attenuate the translation and decrease the protein load within the ER lumen [79]. However, if the stress-induced PERK activation sustains, apoptosis is induced. PERK-mediated apoptosis mainly relies on the enhanced translation of Activating Transcription Factor 4 (ATF4), a member of the CCAAT/Enhancer-Binding Protein (C/EBP) family of transcription factors [80]. ATF4 activates the expression of a pro-apoptotic transcription factor C/EBP Homologous Protein (CHOP) gene [81]. Although CHOP was first discovered to be involved in the cellular response to DNA damage, it was later demonstrated to be a key protein responsible for cell death establishment through two signaling arms. On one hand, CHOP activates the expression of apoptotic and ROS-generation genes such as *Endoplasmic Reticulum Oxidoreductin 1* (*ERO1α*) [82] and *Death Receptor 5* (*DR5*) [83]; on the other hand, CHOP reverses the translational attenuation imposed by PERK via a negative feedback loop [84]. CHOP can also diminish the expression of anti-apoptotic proteins such as Bcl-2 [85]. PERK activation can also induce the expression of miR-30c-2*, a microRNA that suppresses XBP1-mediated gene expression, leading to cell death [86].

Another ER stress sensor ATF6, which is the functional homolog of plant bZIP28, can also control the expression of CHOP and overexpression of ATF6 enhances CHOP expression and promotes apoptosis [87]. Moreover, ATF6 has been demonstrated to trigger cell death in myoblast cells through expression inhibition of an anti-apoptotic protein Myeloid Cell Leukemia sequence 1 (Mcl-1) [88].

In addition to PERK and ATF6, the mammalian IRE1α can also trigger apoptosis via two different pathways. Firstly, IRE1α cytoplasmic domain binds to an adaptor protein Tumor Necrosis Factor Receptor Associated Factor 2 (TRAF2), which activates the apoptosis signaling-regulating kinase 1/c-Jun amino terminal kinase cascade (ASK1/JNK1) [89]. JNK can phosphorylate different family members of Bcl-2 [90], triggering the release of pro-apoptotic BH3-only proteins. JNK can also directly phosphorylate BH3 [91]. Secondly, RIDD can facilitate the degradation of mRNAs encoding proteins crucial for cell viability such as chaperones [92]. Interestingly, the pro-survival protein BAX-inhibitor-1 (BI-1), an Bcl-2 interactor, was also shown to interact with IRE1α in animals and hereby negatively regulate IRE1α activity [93,94].

Although it is classically known that IRE1α cleaves an additional intron out of XBP1 mRNA to promote cell survival [95], a recently identified switch can redirect the RNase activity of IRE1α from XBP1 splicing to specific decay of mRNAs encoding pro-survival proteins [92]. Notably, IRE1 α-mediated TRAF2-JNK-CHOP and RIDD pathways function in concert, which may rely on the conformational changes during IRE1 oligomerization [96].

The three ER stress sensors are not the only link between ER stress and cell death. ER is the main reservoir of Ca^2+^ and dynamic changes of intracellular Ca^2+^ concentration have been implicated in a variety of signaling pathways following activation of different stimuli. ER has a number of close contacts with mitochondria, forming a vigorous inter-organelle communicating network [97]. Upon stress, ER releases Ca^2+^ into mitochondria, leading to the discharge of cytochrome c from the mitochondria into cytoplasm, thereby activating the caspase cascade [97] (Figure 3). In addition, overexpression of BAX and BAK can increase the Ca^2+^-triggered cell death by stimulating cytochrome c release [98]. Depletion of Ca^2+^ in ER also interferes with the function of Ca^2+^-dependent ER-resident chaperones, which can intensify the protein overload, leading to cell death [99].

## 5. Caution: Limited Visibility Ahead—PCD in Plants

PCD is a well-known phenomenon in plants, but a strict categorization of different types of PCD as in the animal system has not been possible to date [100]. Interestingly, homologs of many conserved animal PCD components, such as Bcl-2, BAX/BAK and caspases, are not found in plants. However, caspase-like activity has been detected during PCD in plants and homologs of other components of the Bcl2-BAX/BAK signaling cascade are found in plants, suggesting at least some functional overlaps between plant and animal PCD signaling. PCD can play dual roles in plant immunity. PCD either defends the plant by sealing biotrophic pathogens inside patches of dead tissue, as in the case of the HR or PCD acts as a disadvantage to the host plant as in the case of necrosis induced by necrotrophic pathogens. A few ER resident proteins involved in pathogen-induced PCD signaling cascades have been identified, but the roles of plant ER stress sensors in PCD remain elusive. Below we describe several well demonstrated regulators of PCD in plant-pathogen interactions and highlight the roles of ER-localized proteins. Furthermore, possible roles of plant ER stress sensors in PCD will be discussed.

### 5.1. Caspase-Like Activity and Plant PCD

Although lacking similar cleavage specificities, metacaspases (MC) found in plants are distant homologs of animal caspases. There are three type I and six type II metacaspase genes in the Arabidopsis genome [101]. MC1 was demonstrated to positively regulate cell death observed in *Lesions Simulating Disease 1* (*lsd1*) Arabidopsis mutants and MC1 interacted with LSD1 in planta [102]. In addition, genetic analysis of MC8 supported that it is a positive regulator of oxidative stress triggered by UVC and H_2_O_2_ [103]. Not all of the nine Arabidopsis metacaspases possess a positive regulatory function upon cell death. MC2, another type I metacaspase, negatively regulates the previously discussed AtMC1 and suppresses cell death displayed in the *lsd1* plants [102]. A different plant protein with caspase-1-like activity is Vacuolar Processing Enzyme (VPE) [104,105,106]. Even though VPE was first discovered to be involved in processing seed storage proteins [104,107], it is now evident that VPE plays a role in a wide variety of PCD in response to biotic and abiotic stimuli [108]. It has been shown that VPE is involved in HR [109,110]. In the presence of two synthetic caspase inhibitors (specific for caspase-1 and caspase-3 respectively), HR cell death triggered by a *Pseudomonas syringae* pv. *phaseolicola* strain was greatly diminished [111]. Moreover, TMV-triggered HR cell death was associated with caspase enzymatic activity [111], and VPE was later on proved to be pivotal for the cell death *via* virus-induced gene silencing (VIGS) assay [106,109]. Additionally, some necrotrophic pathogens can take advantage of VPE activity to kill plant cells and acquire nutrients. *Fusarium moniliforme* secretes fumonisin B1 that not only causes diseases in corn [112], but also leads to VPE-dependent cell death in Arabidopsis [110]. Four VPE homologs, αVPE, βVPE, γVPE and δVPE, exist in Arabidopsis among which γVPE contributes the most to the fumonisin B1-induced cell death [110]. Beyond the essential roles VPE plays in the cell death in the interactions between host plants and pathogens, VPE also contributes to the cell death required for the root colonization of a symbiotic fungus, *Piriformospora indica* [113]. In order to colonize Arabidopsis roots, *Piriformospora indica* induces PCD in a VPE-dependent manner and simultaneously blocks pro-survival ER stress signaling [113]. Interestingly, VPE was also demonstrated to be involved in abiotic stress. Two soybean transcription factors *Gm*NAC30 and *Gm*NAC81 coordinately activate PCD by binding to the *VPE* promoter and further enhancing the expression of VPE in response to the tunicamycin-induced ER stress and polyethylene glycol-triggered osmotic stress [114].

### 5.2. Homologs of BAX/BAK and Bcl-2 Interacting Proteins in Plants

Even though the attempts to identify Bcl-2 and BAX/BAK orthologues in plants have failed, homologs of widely conserved Bcl-2 interacting proteins such as BI-1 and Bcl-2-Associated Athanogene (BAG) were identified in plants. The BI-1 protein has been linked to ER stress-triggered apoptosis in mammalian cells. The search of BI homologs in plants led to the discovery of Arabidopsis BI-1 as well as its homologs in barley and rice [115,116]. BI-1 localizes to ER and nuclear membranes [117]. ER stress-induced cell death in Arabidopsis was increased in the absence of BI-1 and consistently, cell death was generally depressed when BI-1 was overexpressed [115].

**Figure 4:**
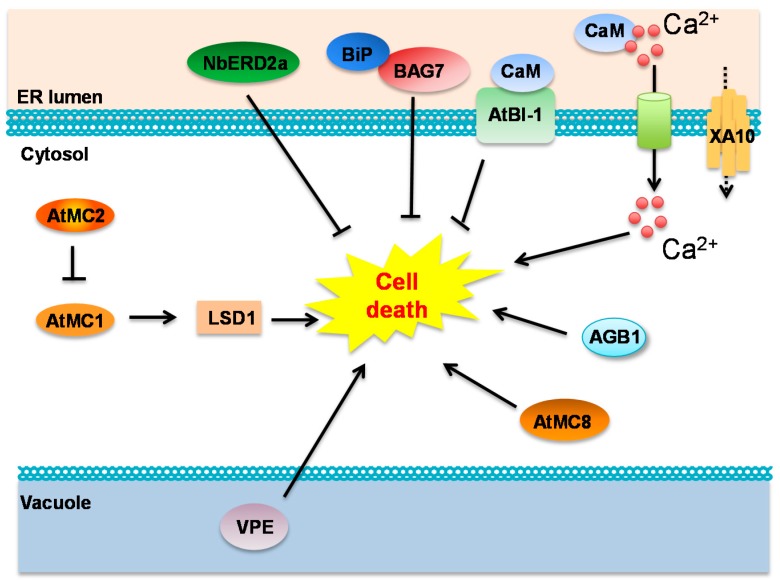
Components involved in plant Programmed Cell Death (PCD). Members of the metacaspase (MC) family can act as both positive and negative regulators of PCD. Vacuolar Processing Enzymes (VPEs) is another group of proteins with caspase-like activity which has been shown to promote pathogen-induced PCD. ER proteins involved in Ca^2+^ signaling such as Ca^2+^ sensor Calmodulin (CaM) and putative Ca^2+^ transporter XA10 also control PCD. ER-resident chaperones Binding Protein (BiP) and Bcl-2-Associated Athanogene 7 (BAG7) acts as negative regulators of PCD, as does the plant homolog of BAX inhibitor-1 (BI-1). The exact molecular mechanism by which these components control and/or interact with each other in the regulation of PCD in plants remains unknown.

BI-1’s role in PCD could be mediated through suppression of ROS production [118], but Arabidopsis BI-1 also interacts with ER-localized Ca^2+^ sensor Calmodulin (CaM) 7, one of seven CaMs encoded by Arabidopsis [117], suggesting a role for BI-1 in Ca^2+^ release from the ER during PCD as is the case for the animal BI-1. As expected, increase in BI-1 activity benefits biotrophic fungal pathogens while hampering necrotrophic fungi [115]. Until now all evidence in plants suggests that BI-1 acts as a negative regulator of cell death (Figure 4), akin to what is observed in animals.

Bcl-2 co-chaperone BAG7 was identified in Arabidopsis [119]. Animal Bcl2-interacting BAGs localize to the cytosol, while BAG7 was shown to associate with BiP2 in the ER lumen. Arabidopsis *bag7* knockout mutants displayed accelerated cell death after treatment with tunicamycin [119] but it remains unknown if the role of BAG7 in PCD depends on a Bcl-2-like mechanism (Figure 4) and the role of BAG7 in pathogen induced PCD has not been addressed yet.

### 5.3. ER-Localized Cell Death Regulators

The retrograde pathway from Golgi back to the ER is important for proper ER function. Two *N. benthamiana* ER Retention Defective (ERD) homologs have been identified as ER luminal protein receptors that successfully retrieved KDEL-tagged GFP [120]. Interestingly, VIGS of *Nb*ERD2a promoted cell death triggered by a variety of elicitors including Xoo, *Pseudomonas syringae* pv. *tomato* DC3000, N-TMV-p50, Cf9-Avr9 and Pto-AvrPto, indicating that *Nb*ERD2a negatively regulates cell death [120].

ER resident proteins BiP and CRT3 were also found to be involved in *Cf*-dependent HR in *N. benthamiana*. Both BiP and CRT3 interact with the R protein Cf and a role of BiP and CRT3 in protein folding and stabilization of the Cf protein has been suggested. This would bear resemblance to what has been observed for some MAMP receptors [121]. A different study also found evidence supporting the role of the ER protein folding machinery in HR. Not only were the levels of BiP and other ER folding proteins found to increase during TMV-induced HR in *N. benthamiana*, but silencing of *CRT2* and *CRT3* decreased HR and allowed viral movement [122]. The authors suggest that incorrect folding and degradation of Induced Receptor-Like Kinase 1, a plasma membrane-localized receptor with unknown function, in CRT-silenced plants could explain the observed phenotype. Overexpression of BiP accelerated non-host *Pseudomonas syringae* pv. *tomato* DC3000-induced HR in soybean [123], once again suggesting a role of BiP as a positive regulator of PCD. However, the dual role of BiPs in protein folding and as regulators of ER stress sensor activity makes it difficult to unequivocally pinpoint the function of this protein in PCD.

Ca^2+^ efflux from the ER lumen into the cytosol is another hallmark of early PCD signaling. Two putative ER-localized Ca^2+^ transporters with a role in pathogen-induced PCD have been identified in plants [124,125]. The rice *R* gene *Xa10* needed for HR resistance against *Xanthomonas oryzae* pv. *oryzae* was recently cloned [125]. *Xa10* encodes is a putative Ca^2+^ transporter localized to the ER membrane and overexpression of XA10 lead to depletion of Ca^2+^ from the ER lumen [125]. In contrast, an ER localized Calcium ATPase (CA1) was identified as negative regulator of PCD through a high-throughput VIGS screen in *N. benthamiana*. The mechanism by which CA1 inhibits HR is still unclear [124].

### 5.4. Plant ER Stress Sensors and PCD

Despite the detailed understanding of the animal ER stress sensors in PCD, little is known about the plant homologs and their role in pathogen induced PCD. In Arabidopsis, infection with an avirulent *Pseudomonas syringae* pv. *maculicola* avrRpm1 strain induced splicing of bZIP60 mRNA, the hallmark of IRE1 activation [60]. However, it is not clear at present if IRE1 and bZIP60 act as negative or positive regulators of HR, and based on observations in animal systems it could be expected that IRE1 plays a dual role in the cell fate regulation. Accelerated cell death after treatment with ER stress inducer tunicamycin in Arabidopsis *ire1a ire1b* double mutants but not the *bzip60-1* mutants led the authors to speculate that IRE1-dependent RIDD is involved in cell death [24]. However, it was not addressed if the cell death induced by high concentrations of tunicamycin is biologically equivalent to PCD and therefore relies on the PCD signaling cascade or, alternatively, if tunicamycin-induced cell death is merely due to acute toxicity.

As described earlier, the G protein β subunit AGB1 plays a role in both ER stress and MAMP signaling pathways. The Arabidopsis *agb1* mutant plants furthermore displayed less severe cell death compared to the wild-type plants after infiltration with tunicamycin [47]. In addition, defense-related cell death triggered in an Arabidopsis *bir1-1* (*BAK1-interacting receptor-like kinase1*) mutant was suppressed by mutations in AGB1 [51].

## 6. Concluding Remarks

In recent years, our understanding of ER stress signaling in plants has been greatly enhanced. Despite accumulating evidence that links ER stress signaling with immunity, the role of the different ER stress signaling components in immunity is still largely unexplored. The importance of plant IRE1 homologs for regulating of ER stress responses is well established, but experimental evidence for the molecular mechanism activating IRE1 is still lacking. It will be interesting to see if IRE1 activation by ER stress-inducing chemicals such as tunicamycin can be considered equivalent to IRE1 activation by biotic and abiotic stresses [33]. Moreover, IRE1 has been shown to be involved in plant immunity [60] and considering the partially overlapping roles of IRE1 and bZIP28 in chemically induced ER stress, a possible involvement of bZIP28 in immunity cannot be ruled out. Further studies will be needed to answer this question. Another open avenue for future research is the role of ER stress signaling in plant PCD. PCD, and in particular HR, plays an important role in plant immunity and it will be interesting to see if IRE1 and bZIP28 are involved in regulating PCD similar to what is described for their animal homologs, IRE1α and ATF6.
